# MAGE-Targeted Gold Nanoparticles for Ultrasound Imaging-Guided Phototherapy in Melanoma

**DOI:** 10.1155/2020/6863231

**Published:** 2020-09-18

**Authors:** Xuelin Li, Shigen Zhong, Cuncheng Zhang, Pan Li, Haitao Ran, Zhigang Wang

**Affiliations:** ^1^Department of Geriatric, Chongqing General Hospital (University of Chinese Academy of Sciences Chongqing Hospital), Chongqing 400014, China; ^2^Department of Ultrasound, Chongqing General Hospital (University of Chinese Academy of Sciences Chongqing Hospital), Chongqing 400014, China; ^3^Institute of Ultrasound Imaging of Chongqing Medical University, Chongqing Key Laboratory of Ultrasound Molecular Imaging, Chongqing 400010, China

## Abstract

Gold nanorods exhibit a wide variety of applications such as tumor molecular imaging and photothermal therapy (PTT) due to their tunable optical properties. Several studies have demonstrated that the combination of other therapeutic strategies may improve PTT efficiency. A method called optical droplet vaporization (ODV) was considered as another noninvasive imaging and therapy strategy. Via the ODV method, superheated perfluorocarbon droplets can be vaporized to a gas phase for enhancing ultrasound imaging; meanwhile, this violent process can cause damage to cells and tissue. In addition, active targeting through the functionalization with targeting ligands can effectively increase nanoprobe accumulation in the tumor area, improving the sensitivity and specificity of imaging and therapy. Our study prepared a nanoparticle loaded with gold nanorods and perfluorinated hexane and conjugated to a monoclonal antibody (MAGE-1 antibody) to melanoma-associated antigens (MAGE) targeting melanoma, investigated the synergistic effect of PTT/ODV therapy, and monitored the therapeutic effect using ultrasound. The prepared MAGE-Au-PFH-NPs achieved complete eradication of tumors. Meanwhile, the MAGE-Au-PFH-NPs also possess significant ultrasound imaging signal enhancement, which shows the potential for imaging-guided tumor therapy in the future.

## 1. Introduction

Melanoma is a malignant neoplasm sourced from melanocytes skin cells—with poor prognosis at advanced stages. Standard cancer treatments can be highly toxic to healthy tissues without differentiating malignant from normal cells, causing significant adverse effects in patients. Nanoparticle-based photothermal ablation therapy assisted by near-infrared (NIR) laser holds a promise to eliminate tumors noninvasively, reduce tumor resistance, and prevent recurrence [1–3]. In addition, active targeting ability through functionalization with specific ligands can effectively enable nanoprobes to accumulate in the tumor area [2]. Melanoma-associated antigens (MAGE) are a specific and highly expressed family of antigens in malignant melanoma [4–6]. Therefore, MAGE proteins could also be used to functionalize nanoprobes for molecular imaging and accurate treatment of melanoma.

Among the available nanoparticle systems, gold nanorods (GNRs/Au-NRs) have attracted particular attention in cancer imaging and photothermal therapy [7–9] due to several advantages: biocompatibility, high photothermal conversion efficiency, well-established methods for synthesis in a wide range of sizes, and ease of biomodification [10]. However, complete tumor eradication is so far difficult to achieve with the introduction of these photothermal nanomaterials. In particular, their low tissue bioabsorption and utilization result in limited curative effect in deeply located tumors [11]. Recently, it has been reported that PTT efficiency may be improved with the combination of other therapeutic strategies [12–14]. Phase-changeable liquid fluorocarbon emulsions can be vaporized and transformed from droplets to microbubbles under optical irradiation (ODV) [15–18] which has become an attractive noninvasive theranostic protocol based on ultrasound imaging and physical therapy [19]. The emulsion significantly increased acoustic impedance between the tumor and surrounding tissues [20] and causes physical damage to tumor cells [21].

Poly(D,L-lactide-co-glycolide) (PLGA), as a type of biodegradable nanomaterial with an excellent safety profile in humans [22] and good film-forming ability, has been approved by FDA for vaccine and drug delivery as well as tissue engineering [23–25]. Thus, we prepared a MAGE-targeted PLGA nanomolecular probe encapsulating liquid perfluorohexane (PFH) and Au-NRs (MAGE-Au-PFH-NPs) in this work, specifically targeting melanoma cells, which have been confirmed to possess the potential to enhance photoacoustic (PA) and ultrasonic imaging in melanoma in our previous study. The results from our previous study showed that MAGE-Au-PFH-NPs could accumulate and retain in the tumor area, allowing for therapeutic guidance and monitoring [26]. This study went further in exploring the role of the MAGE-Au-PFH-NPs in the treatment of melanoma based on our previous study. Benefiting from the high photothermal-conversion efficiency and ODV effect in MAGE-Au-PFH-NPs, the efficient tumor ablation was achieved in melanoma-bearing mice, which provides a promising alternative strategy for imaging-guided phototherapy of cancer.

## 2. Materials and Methods

### 2.1. Materials

Gold nanorods (Au-NRs, 780 nm) were purchased from NanoSeedz Ltd. (Hong Kong SAR), perfluorohexane (PFH) was from Ji'nan Daigang Biological Engineering Co. Ltd. (Jinan, China), and the B16 mouse melanoma cell line was purchased from the Punuosai Company (Wuhan, China). MAGE-1 antibody was from the Bioye Company (Shanghai, China), and propidium iodide (PI) was purchased from Sigma-Aldrich (St. Louis, MO, USA). Calcein acetoxymethyl ester (Calcein-AM) was purchased from Santa Cruz Biotechnology (TX, USA). Anti-HSP70 Rabbit pAb was from Servicebio (Wuhan, China). Cy3-conjugated goat anti-rabbit IgG was from Servicebio (Wuhan, China).

### 2.2. Cell Culture and Animal Experiment

B16 mouse melanoma cells were cultured in T75 flasks containing Roswell Park Memorial Institute (RPMI) 1640 medium supplemented with 10% foetal bovine serum and 1% penicillin and streptomycin (antibiotics) and incubated at 37°C under 5% CO_2_, with medium changes every 2-3 days. For all the experiments, the cells were harvested using 0.25% trypsin solution and were then resuspended in fresh medium before plating.

All the animals (male BALB/c nude mice: ~20 g, 4–6 weeks) were purchased from the Experimental Animal Center of Chongqing Medical University and bred at constant temperature and humidity, with food and water provided *ad libitum*. The animals were maintained in accordance with the National Guidelines for Experimental Animal Welfare (MOST, China, 2006) at the Centre for Animal Experiments, and all the experiments and procedures were approved by the Institutional Animal Care and Use Committee of Chongqing Medical University. B16 cells were suspended in PBS (1 × 10^6^ B16 cells in 100 *μ*L of PBS per mouse) and then injected subcutaneously into the buttock of the BALB/c nude mice to establish tumor-bearing mice.

### 2.3. Characterization of the NPs

By reference to our previous study [26], MAGE-Au-PFH-NPs were prepared by the double emulsion method. The carbodiimide method was employed to modify the Au-PFH-NPs with MAGE antibody to prepare the targeted nanoparticles (MAGE-Au-PFH-NPs).

The prepared NPs were observed under a confocal laser scanning microscope (Nikon) and transmission electron microscope (TEM) (H-7500; Hitachi, ×1.5 k Zoom-1 HC-1 80.0 kv).

The size and zeta potential of MAGE-Au-PFH-NPs were measured by a Malvern laser particle size analyzer (Malvern, England). Furthermore, to assess the stability of MAGE-Au-PFH-NPs, the NP size and zeta potential changes were tested for 72 hours while incubating in plasma at 37°C. Meanwhile, the NPs underwent PAI scanning at different wavelengths ranging from 680 nm to 970 nm (interval = 5 nm) to determine the maximum absorbance for optimized PAI by a Vevo LAZR Photoacoustic Imaging System (Vevo LAZR, Toronto, Canada).

### 2.4. *In Vitro* Photothermal Properties of NPs

In our previous study, MAGE-Au-PFH-NP temperature was increased to 70°C through photothermal conversion after absorbing near infrared light [26]. In this study, we evaluated photothermal capability of NPs in different concentrations at different power densities by laser irradiation using an 808 nm laser for 5 minutes *in vitro*. The infrared radiation (IR) thermal images and temperature changes were recorded by an infrared thermal-imaging camera.

### 2.5. *In Vitro* Photothermal Ablation against B16 Cells

To test the photothermal ablation effects of MAGE-Au-PFH-NPs *in vitro*, B16 cancer cells were divided into 4 groups: control (normal saline (NS) only), NPs only, laser only, and NPs+laser, and were seeded onto four confocal-specific cell-culture dishes (1 × 10^5^ cells per dish) overnight. After cell adhesion, NP suspension was added into two dishes (two groups: NPs only and NPs+laser), and equal volume serum-free RPMI 1640 medium was added into the other two dishes and incubated for another 12 h; cells of two groups (laser only and NPs+laser) were exposed to laser (1.00 W/cm^2^) for 10 min. Then, the medium was removed, and the cells of each dish were washed three times with PBS. The cells of four groups were scanned by confocal microscopy after costaining with Calcein-AM and propidium iodide.

### 2.6. Photothermal Conversion Evaluation and Heat Shock Protein (HSP) Evaluation *In Vivo*

To investigate the *in vivo* photothermal efficiency of MAGE-Au-PFH-NPs, twenty B16 tumor-bearing mice were randomly divided into four groups (5 mice per group) when the tumor volumes reached about 100 mm^3^, including MAGE-Au-PFH-NP, Au-PFH-NP, Au-NP, and NS groups. The mice were intravenously injected with 100 *μ*L MAGE-Au-PFH-NPs, Au-PFH-NPs, and Au-NPs at a concentration of 25 mg/mL, respectively. The same volume of normal saline was injected into the mice in the control NS group. The tumors were exposed to the 808 nm laser (1.00 W/cm^2^) for 10 min after injection of NPs and NS 2 minutes later. The temperature changes in tumors and infrared radiation (IR) thermal images were recorded by an infrared thermal-imaging camera. To further analyze the effect of photothermal therapy on local hyperthermia, we detected the HSP70 expression within tumors by immunofluorescent staining. All the dose and laser irradiation conditions were adjusted to the same level as mentioned above. At the second day after treatment, mice were sacrificed to collect tumors for HSP70 immunofluorescent staining. Anti-HSP70 rabbit pAb as primary antibodies was added in the section and incubated overnight at -4°C; Cy3 conjugated goat anti-rabbit IgG as secondary antibodies was added in the section away from light for 50 min at room temperature, followed by DAPI hyperchromatic nucleus and sealing piece. Eventually, the sections were observed and imaged under a fluorescence microscope.

### 2.7. Targeting Ability *In Vitro* and *In Vivo*

To assess the targeting ability *in vitro*, immunofluorescence imaging observed under confocal microscopy has been performed. The melanoma-associated antigens were combined to the targeted nanoparticles to obtain the blocking group (MAGE-R-Au-PFH-NPs). MAGE-Au-PFH-NPs and the blocking group (MAGE-R-Au-PFH-NPs) were all treated with DiI fluorescent dye in the first step of synthesis before the sonication. B16 cells were seeded in confocal laser dishes at 1 × 10^5^ and coincubated with dyed NPs (MAGE-Au-PFH-NPs, MAGE-R-Au-PFH-NPs) at 37°C for 30 min and then observed under a laser scanning confocal microscope after being fixed with 4% paraformaldehyde and stained with 20 *μ*L of 4′,6-diamidino-2-phenylindole (DAPI).

To detect whether MAGE-Au-PFH-NPs have a long circulation time compared to that of the Au-PFH-NPs, 6 tumor-bearing mice were randomly divided into two groups (MAGE-Au-PFH-NPs and Au-PFH-NPs); the two groups of mice were intravenously injected with 100 *μ*L of MAGE-Au-PFH-NPs and Au-PFH-NPs (25 mg/mL). The dynamic distribution of nanoparticles within the whole body was measured using fluorescence spectrum.

### 2.8. Photothermal/ODV Efficiency and Detection *In Vivo*

To evaluate the *in vivo* photothermal and ODV efficiency of MAGE-Au-PFH-NPs, twenty B16 tumor-bearing mice were used when the tumor volumes reached about 100 mm^3^. The mice were divided into four groups (5 mice per group) randomly including the laser only, Au-NP+laser, Au-PFH-NP+laser, and MAGE-Au-PFH-NP+laser groups, which were intravenously injected with normal saline solution (100 *μ*L), Au-NP (25 mg/mL, 100 *μ*L), Au-PFH-NP (25 mg/mL, 100 *μ*L), and MAGE-Au-PFH-NP suspension (25 mg/mL, 100 *μ*L), respectively. Then, each mouse was exposed to the 808 nm laser for 10 min at a power density of 1.00 W/cm^2^. The treatment was performed every other day, and one of the mice in each group was sacrificed on the third day for pathological section and staining. All the tumor tissues were collected and fixed in a 4% paraformaldehyde solution, stained with H&E, TdT-mediated dUTP Nick-End Labeling (TUNEL), and Proliferating Cell Nuclear Antigen (PCNA) for histopathology analysis.

The remaining four mice were maintained for 15 days, and the mouse weight and tumor volume were measured every other day after PTT. The tumor-volume changes were normalized using the relative tumor volumes *V*/*V*_0_ (*V*_0_: the initial tumor volume before the treatment).

In our previous study, CEUS imaging was significantly enhanced at the tumor site in the MAGE-Au-PFH-NP group after laser irradiation [26]. Thus, contrast-enhanced ultrasonography (Esaote MyLab 90, Genoa, Italy) was performed in tumor-bearing mice after the treatment to evaluate the therapeutic effect of MAGE-Au-PFH-NPs *in vivo* in this study. CEUS-mode images of the tumors were recorded after exposure to the 808 nm laser (1.00 W/cm^2^, 10 mins). Echo intensity was acquired and analyzed using a DFY-ultrasonic image quantitative analyzer (Institute of Ultrasound Imaging of Chongqing Medical University, Chongqing, China).

### 2.9. Toxicity Test *In Vitro* and Biocompatibility *In Vivo*

To assess the toxicity of MAGE-Au-PFH-NPs, the typical CCK-8 assay was used to evaluate the cell viability in endothelial cells and hepatic cells. The test of liver functional markers (AST, ALT), kidney functional markers (CR, BUN), and H&E staining of the major organs (heart, liver, and kidney) after intravenous injection and laser irradiation have been performed to evaluate the biocompatibility *in vivo*.

## 3. Statistical Analysis

Data were presented as the mean ± standard deviation. Statistical analyses were done using the SPSS Ver. 19.0. *P* < 0.05 was considered statistically significant according to one-way ANOVA and Student's *t*-test.

## 4. Results

### 4.1. Characterization of NPs and Photothermal Conversion Efficiency of NPs *In Vitro*

**Figure 1:**
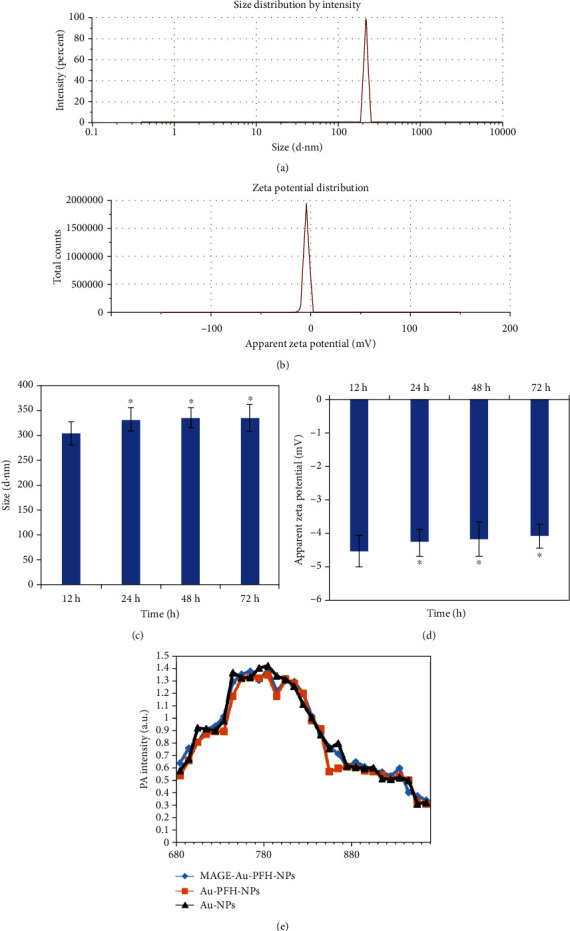
Physicochemical characterization of MAGE-Au-PFH-NPs. (a) Size distribution of MAGE-Au-PFH-NPs. (b) Zeta potential of MAGE-Au-PFH-NPs. (c) Size distribution of MAGE-Au-PFH-NPs incubating in plasma at 37°C after 12, 48, and 72 h (^∗^*P* > 0.05). (d) Zeta potential of MAGE-Au-PFH-NPs incubating in plasma at 37°C after 12, 48, and 72 h (^∗^*P* > 0.05). (e) PA spectrum of MAGE-Au-PFH-NPs, Au-PFH-NPs, and Au-NPs (25 mg/mL) from 680 nm to 960 nm.

The size and zeta potential of MAGE-Au-PFH-NPs were 324.54 ± 21.76 nm and −4.76 ± 3.7 mV, respectively, measured by a Malvern laser particle size analyzer. Furthermore, results showed that the size and zeta potential changes measured at 48 h and 72 h had no statistic difference compared with 12 h that could confirm the stability of MAGE-Au-PFH-NPs (Figures 1(a)–1(d)). The result of the PAI scanning showed that the maximum absorbance of three groups (Au-NPs, Au-PFH-NPs, and MAGE-Au-PFH-NPs) had been detected at 780 nm which conformed to the spectral properties of gold nanorods (GNRs/Au-NRs) (Figure 1(e)).

**Figure 2:**
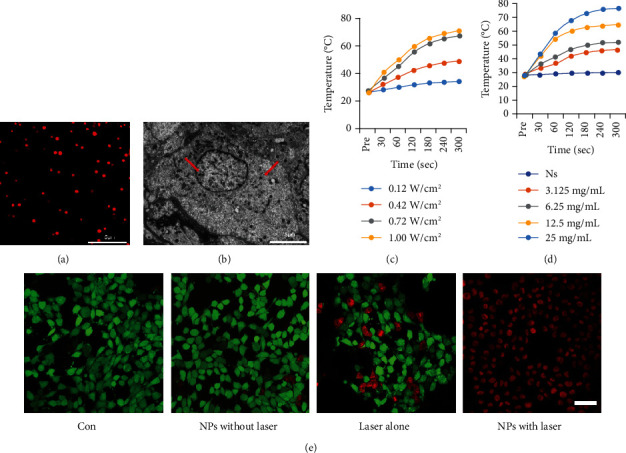
Morphological distribution of MAGE-Au-PFH-NPs under the microscope and photothermal properties of NPs *in vitro*. (a) CLSM image of DiI-stained MAGE-Au-PFH-NPs (scale bar: 5 *μ*m). (b) TEM image of MAGE-Au-PFH-NP distributions in cells (scale bar: 5 *μ*m). Red arrow showed the NP distribution in B16 cells. (c) Plot of temperature change of MAGE-Au-PFH-NP suspension at different power densities of an 808 nm laser (0.12, 0.42, 0.72, and 1.00 W/cm^2^) as a function of irradiation duration (MAGE-Au-PFH-NP concentration: 25 mg/mL and 100 *μ*L). (d) Plot of temperature change of NS and MAGE-Au-PFH-NP suspension at different levels of concentration (MAGE-Au-PFH-NP concentrations: 3.125, 6.25, and 13.5, 25 mg/mL and 100 *μ*L) exposure to an 808 nm laser (1.00 W/cm^2^) as a function of irradiation duration. (e) Confocal fluorescence imaging of Calcein-AM and PI costained B16 cells after coincubation with NPs for 12 h followed by different treatments (scale bar = 100 *μ*m).

The NPs showed a good dispersity under a confocal laser scanning microscope and were mainly distributed in the cytoplasm after uptake by cells under a transmission electron microscope (Figures 2(a) and 2(b)).

The temperature change of NPs after laser irradiation was recorded by an infrared thermal-imaging camera. The temperature in a MAGE-Au-PFH-NP suspension increased significantly and reached as high as 71°C under NIR irradiation within 5 min at a concentration of 25 mg/mL as shown in Figures 2(c) and 2(d), while no temperature change was found in the normal saline (NS) group. Moreover, the temperature elevated significantly with the increase of NIR irradiation power, which demonstrated that the photothermal conversion efficiency of MAGE-Au-PFH-NPs depended on both concentration and laser power density.

### 4.2. *In Vitro* Photothermal Ablation against B16 Cells

A large number of B16 cells died and showed a strong red fluorescence (Figure 2(e)) in the MAGE-Au-PFH-NP+laser group in the experiment *in vitro*, suggesting photothermal effect induced by MAGE-Au-PFH-NPs under external NIR irradiation. In contrast, dead cells were rarely found in the control group, which displayed green fluorescence of Calcein-AM staining (Figure 2(e)). Only a number of dead cells were shown in the laser- and MAGE-Au-PFH-NP only groups, as confirmed by the strong green fluorescence and very weak red fluorescence from PI staining.

### 4.3. Photothermal Conversion Efficiency and HSP Evaluation *In Vivo*

**Figure 3:**
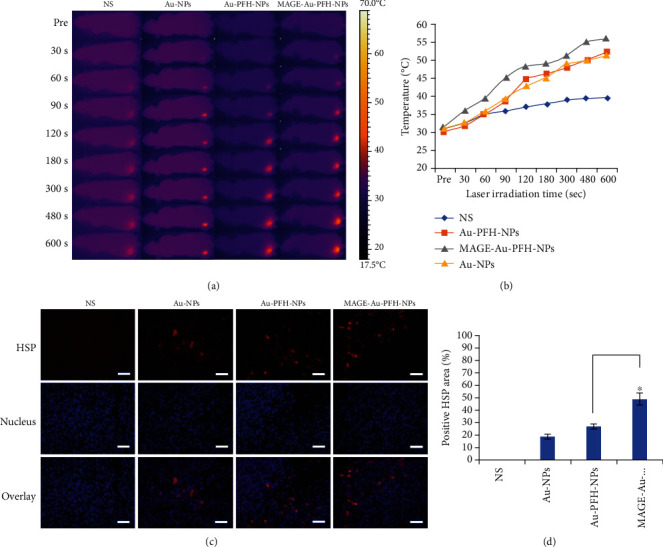
Photothermal conversion efficiency and heat shock protein (HSP) evaluation *in vivo*. (a) IR thermal images of B16 tumor-bearing mice of the four groups (NS+laser, Au-NP+laser, Au-PFH-NP+laser, and MAGE-Au-PFH-NP+laser groups) taken at different times. (b) Plot of temperature change of four groups (NS+laser, Au-NP+laser, Au-PFH-NP+laser, and MAGE-Au-PFH-NP+laser groups) *in vivo*. (c) Immunofluorescent staining of HSP70 of tumor tissues dissected from different groups on the 1st day post treatments. The scale bar is 50 *μ*m. (d) Quantitative analysis of HSP expression from different groups on the 1st day post treatments. (The data is shown as mean ± SD, *n* = 5 per group; ^∗^*P* < 0.05.).

As shown in Figures 3(a) and 3(b), the surface temperature of tumors in the MAGE-Au-PFH-NP+laser group increased from 31.6 ± 1.09°C to 56.1 ± 2.6°C under irradiation for 10 min. The Au-PFH-NP+laser group increased from 30.1 ± 1.3°C to 52.0 ± 2.1°C under irradiation for 10 min. The temperature in the Au-NP+laser group increased from 31.2 ± 0.9°C to 51.4 ± 1.7°C under irradiation for 10 min. Comparatively, only a slight temperature increase was found in the tumor region in the laser only group.

The HSP70 expression level was analyzed and is shown in Figures 3(c) and 3(d). The mice that received MAGE-Au-PFH-NPs plus laser irradiation showed a remarkably higher HSP70 expression compared to those treated with Au-PFH-NPs and Au-NPs followed by laser irradiation. In the control group (NS with laser irradiation), no evident expression of HSP70 was found.

### 4.4. Targeting Ability *In Vitro* and *In Vivo*

**Figure 4:**
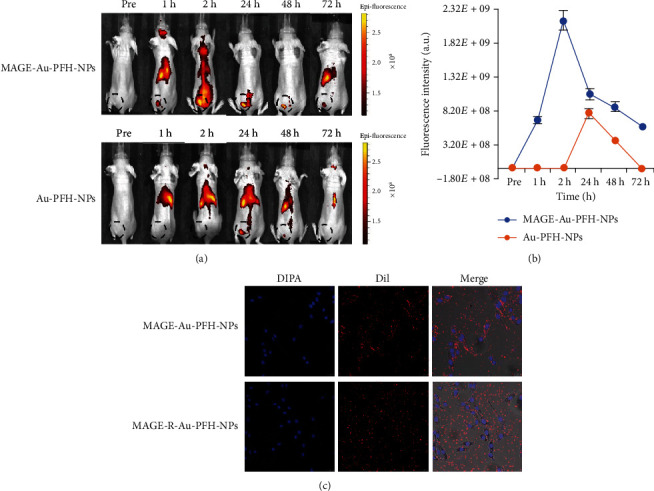
Fluorescence imaging *in vivo*. (a) Fluorescence images of B16 tumor-bearing mice after intravenous injection of MAGE-Au-PFH-NPs and Au-PFH-NPs at 0, 1, 2, 24, 48, and 72 h (*n* = 3 per group). (b) Plot of fluorescence images of B16 tumor-bearing mice after intravenous injection of MAGE-Au-PFH-NPs and Au-PFH-NPs at 0, 1, 2, 24, 48, and 72 h. The dotted circle instruction for the tumor mass. (c) Targeting ability of MAGE-Au-PFH-NPs and MAGE-R-Au-PFH-NPs to B16 cells observed under CLSM; the left line was cell nuclei stained by DAPI, the middle line was NP stained by DiI, and the right line was the merged result of the two fluorescence images.

The biodistribution of nanoparticles in mice was investigated by *in vivo* fluorescence imaging. At 1 hour post injection, prominent uptake of nanoparticles in the tumor was observed in the MAGE-Au-PFH-NP group; the signals reached a peak 2 hours later and lasted for 72 h. While in the Au-PFH-NP group, fluorescent signals were found at 24 h and disappeared after 48 h (Figures 4(a) and 4(b)). These results confirmed the accumulation and long retention time of MAGE-Au-PFH-NPs within the tumor area. Fluorescence imaging observed under confocal microscopy demonstrated a large number of MAGE-Au-PFH-NPs concentrated in B16 cells, showing clear red fluorescence, while the blocking group (MAGE-R-Au-PFH-NPs) presented sparse distribution around the cells (Figure 4(c)). These results confirmed that the MAGE-Au-PFH-NPs could specifically target B16 cells.

### 4.5. Photothermal/ODV Efficiency and Detection *In Vivo*

**Figure 5:**
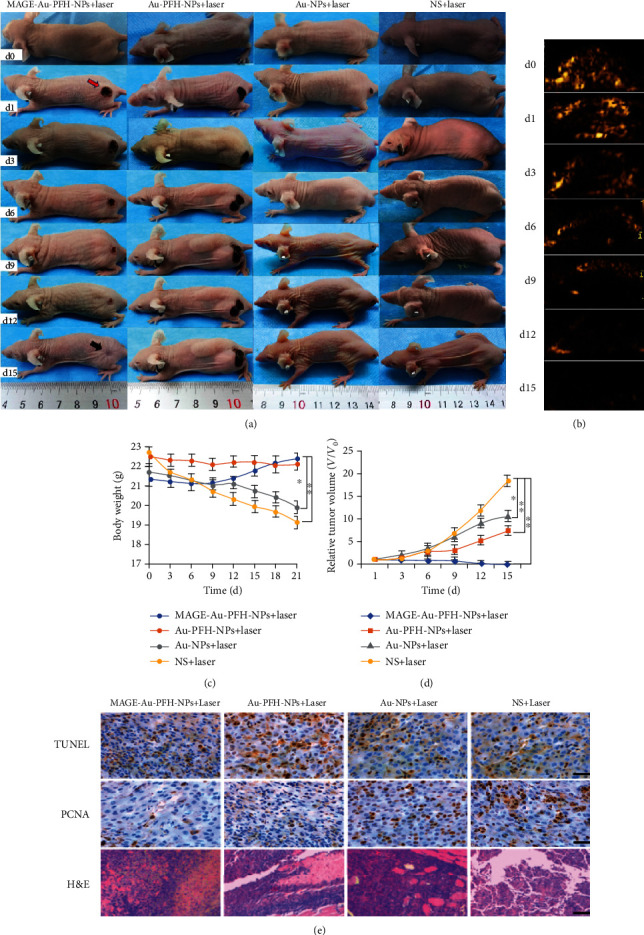
Detection of photothermal/ODV efficiency *in vivo*. (a) Photographs of B16 tumor-bearing mice in the four groups taken during a 15-day period after the various treatments. (b) Ultrasound imaging from the region of interest in B16 tumor-bearing nude mice in the MAGE-Au-PFH-NP group using CEUS after laser irradiation during a 15-day period. (c) Body weight curves (*n* = 5, mean ± SD) of the four groups after different treatments. (d) Tumor growth curves (*n* = 5, mean ± SD) of the four groups after various treatments. (e) H&E, TUNEL, and PCNA staining on tumor sections after various treatments. All the scale bars are 50 *μ*m. (The data is shown as mean ± SD, *n* = 5 per group; ^∗^*P* < 0.05, ^∗∗^*P* < 0.01.).

Based on the *in vivo* photoacoustic imaging experiment, MAGE-Au-PFH-NPs actively accumulated in the tumor region, and the signal reached a peak at 2 h post injection [26]. Therefore, the mice were treated for 10 min after 2 h post injection until the 5th day. Tumor tissue necrosis was found in mice in the MAGE-Au-PFH-NP+laser group the second day after treatment, leaving black scars in the initial tumor regions (Figure 5(a), red arrow). Then, they disappeared 15 days later, leading to complete tumor eradication (Figure 5(a), black arrow), while the tumors in the remaining three groups grew rapidly. The ultrasound imaging was significantly enhanced at the tumor site in the MAGE-Au-PFH-NP group under the laser irradiation. Then, the enhanced ultrasound signals gradually decreased with the increase of treatment times and disappeared by day 15 (Figure 5(b)). The weight and tumor volume of each mouse were recorded every other day (Figure 5(c)). Then, the tumor-volume change was normalized as *V*/*V*_0_ (Figure 5(d)).

H&E and TUNEL staining results further confirmed tumor necrosis in mice in the MAGE-Au-PFH-NP+laser group, which was more serious compared to those in the remaining three groups (Figure 5(e)). From the result of the PCNA assay, the MAGE-Au-PFH-NP+laser group exhibited a significant suppression effect on tumor cell proliferation. In contrast, no evident proliferative inhibition was observed in the remaining three groups.

### 4.6. Toxicity Test *In Vitro* and Biocompatibility *In Vivo*

**Figure 6:**
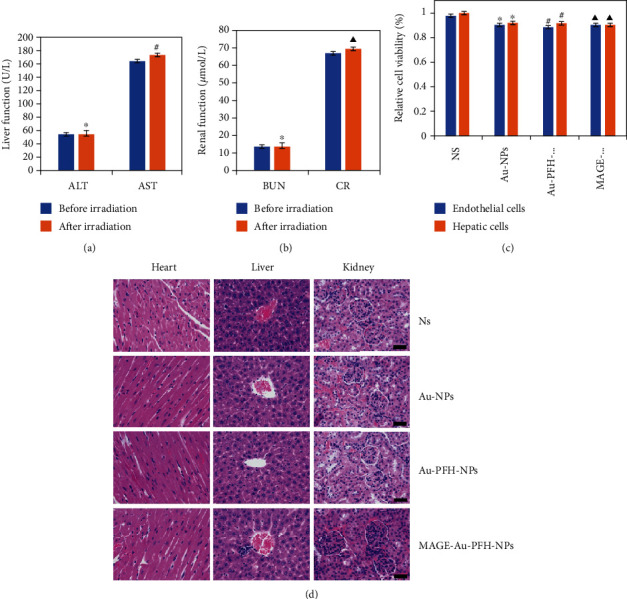
Toxicity test *in vitro* and biocompatibility *in vivo*. (a, b) Haematological assay of B16 tumor-bearing nude mice. (c) Cell viability assay of toxicity of MAGE-Au-PFH-NPs to normal cell (^∗^*P* > 0.05, ^#^*P* > 0.05, and ^▲^*P* > 0.05). (d) H&E staining of the major organs (heart, liver, and kidney) of B16 tumor-bearing nude mice after administration of MAGE-Au-PFH-NPs.

The cytotoxicity of MAGE-Au-PFH-NPs was investigated by the CCK-8 assay. After 24 h incubation, inconspicuous cytotoxicity of the NPs on endothelial cells and hepatic cells was observed since cell viability remained above 80% at NP concentration of 25 mg/mL. And no significant difference was found among the groups (NS, Au-NPs, Au-PFH-NPS, and MAGE-Au-PFH-NPs) (Figure 6(c)). The test of liver functional markers (AST and ALT) and kidney functional markers (CR and BUN) and H&E staining of the major organs (heart, liver, and kidney) after intravenous injection and laser irradiation showed no significant physiological toxicity (Figures 6(a), 6(b), and 6(d)).

## 5. Discussion

Photothermal therapy (PTT) is a minimally invasive technique for cancer treatment which uses laser-activated photoabsorbers to convert photon energy into heat sufficient to induce cell destruction via apoptosis, necroptosis, and/or necrosis. From the current studies, photothermal therapy cannot ablate the tumor thoroughly using photothermal materials due to nonuniform tumor internal heat distribution. The combination of PTT and other methods may overcome this disadvantage. Gold NPs (Au-NPs) designed to act as photothermal sensitizing agents are widely used in cancer therapy due to their high optical absorption coefficients. In our previous study, the Au-NRs and PFH were encapsulated in a PLGA shell through the double emulsion method with a high encapsulation efficiency and conjugated with the MAGE-1 antibody for targeting melanoma cells [26]. With the application of MAGE-Au-PFH-NPs, the temperature can be raised up to 71°C under laser irradiation, showing their excellent photothermal effect [27]. These targeted nanoparticles actively accumulated in the tumor area and enhanced the PTT effect. In addition, the PFH can be vaporized by laser irradiation [28] with the application of MAGE-Au-PFH-NPs [16], which had also been confirmed in our *in vitro* study [26]. Meanwhile, from the *in vivo* results, the temperature in the tumor region increased up to 56.1 ± 2.6°C (Figure 3(b)), which was sufficient to ablate the tumor tissue [29]; meanwhile, it could convert MAGE-Au-PFH-NPs into bubbles. Then, the physical and mechanical damage induced by the phase change process can directly kill tumor cells [21].

Heat shock protein is produced under the induction of stress agents such as high temperature to induce thermoresistance for cells [30]. In our study, the results showed that HSP70 expression in the MAGE-Au-PFH-NP group was significantly higher compared to that in the groups of Au-PFH-NPs and Au-NPs with laser irradiation. Only little expression of HSP70 was found in the NS plus laser group. The results of HSP70 immunofluorescent staining corresponded well with those of photothermal conversion effect.

In our study, complete tumor eradication was observed in the MAGE-Au-PFH-NP+laser group. In contrast, the tumors of the other three groups grew rapidly. The results showed that the combination of targeted photothermal therapy and ODV phase transition physical therapy could achieve better tumor ablation effect. *In vivo* fluorescence imaging confirmed that targeted nanoparticles (MAGE-Au-PFH-NPs) had a longer circulation time compared to the nontargeted Au-PFH-NPs.

Tumor recurrence was found in the Au-PFH-NP and Au-NP plus laser therapy groups indicating limited PTT effect leading to residues in tumor tissue. By combining the PTT effect and ODV physical damage from MAGE-Au-PFH-NPs, the tumor ablation was greatly improved. Meanwhile, damage to normal tissues caused by long time laser irradiation can be avoided.

Besides, when the phase-changeable nanoparticles were transformed from droplets to microbubbles, the acoustic impedance of the tumor and surrounding tissues was increased, which thereby enhanced ultrasound imaging. In this study, the CEUS imaging was significantly enhanced at the tumor site in the MAGE-Au-PFH-NP group under laser irradiation and decreased with the increase of treatment times, providing a method for monitoring tumor ablation effect by CEUS.

## 6. Conclusion

We successfully constructed MAGE-targeted phase-changeable gold nanoparticles which could accumulate at tumor sites. With the combination of PTT and ODV effects, complete tumor eradiation was achieved and could be monitored by contrast-enhanced ultrasonography. Several advantages such as noninvasiveness, short recovery time, low complication rate, and monitorable treatment process were included with this protocol. These novel targeted nanoparticles could be used as a multifunctional theranostic agent for imaging-guided tumor ablation.

## Data Availability

The data used to support the findings of this study are available from the corresponding author upon request.
